# Characterising Correlations between Electric Conductivity and Structural Features in Rotary Swaged Al/Cu Laminated Conductors

**DOI:** 10.3390/ma15031003

**Published:** 2022-01-27

**Authors:** Lenka Kunčická, Radim Kocich, Petr Kačor, Michal Jambor, Martin Marek

**Affiliations:** 1 Institute of Physics of Materials, Czech Academy of Sciences, Žižkova 22, 616 00 Brno, Czech Republic; kuncicka@ipm.cz (L.K.); jambor@ipm.cz (M.J.); 2 Faculty of Materials Science and Technology, VŠB–Technical University of Ostrava, 708 00 Ostrava-Poruba, Czech Republic; 3 Department of Electrical Power Engineering, VŠB–TU Ostrava, 17. listopadu 2172/15, 708 00 Ostrava-Poruba, Czech Republic; petr.kacor@vsb.cz; 4 Department of Technical Studies, College of Polytechnics Jihlava, Tolsteho 16, 586 01 Jihlava, Czech Republic; martin.marek@vsb.cz

**Keywords:** rotary swaging, aluminium, copper, composite, microstructure, electric properties

## Abstract

This study aims to characterize the correlations between electric characteristics and selected structural features of newly designed Al/Cu laminated conductors manufactured via room temperature rotary swaging. After swaging, the laminates with diameters of 15 mm were subjected to two different post-process annealing treatments. Structure analyses performed to evaluate the effects of thermomechanical processing were performed via scanning and transmission electron microscopies. Electric conductivity and resistivity of the laminates were experimentally measured and numerically simulated using models designed according to the real conditions. The results showed that the electric resistivity was affected by the grain size, bimodal grains’ distribution (where observed), the presence of twins, and, last but not least, dislocation density. Among the influencing factors were the area fractions of Al and Cu at the cross-sections of the of the laminated conductors, too. The results revealed that fabrication of the laminate via the technology of rotary swaging introduced more advantageous combinations of electric and mechanical properties than fabrication by conventional manufacturing techniques. The lowest specific electric resistivity of 20.6 Ωm × 10^−9^ was measured for the laminated conductor subjected to the post-process annealing treatment at 350 °C, which imparted significant structure restoration (confirmed by the presence of fine, equiaxed, randomly oriented grains).

## 1. Introduction

Generally, laminates are structures consisting of more than one material (element, alloy), and can be fabricated in various forms (layers, imposed fibres, wires, fibres embedded in resin, etc.). Metallic laminates, which can also be denoted as bimetallic composite materials, are popular modern industrial materials and can find their use e.g., in automotive, aerospace, and marine fields [1,2]. The fact that each of the components introduces specific advantageous properties enhances the performance of the final product.

Not only the design of the laminate, i.e., the selection of component metals and their localization, but also the production method and selected processing temperatures non-negligibly influence the final properties. Metallic laminates can be manufactured by methods (locally) introducing elevated temperatures, such as various types of welding and cladding [3,4,5,6,7]. However, such methods disadvantageously affect structures of the metallic components and promote the formation of brittle intermetallics. For this reason, the fabrication of metallic laminates via methods of intensive (and severe) plastic deformation, which can advantageously be performed at room temperature, is promising.

The methods of plastic deformation are based on imposing (high) shear strain into the processed materials, which enables metallurgical bonding of the individual composite components. Generally, the higher the amount of the imposed strain, the better the bonding quality. For this reason, the methods of severe plastic deformation (SPD), especially equal channel angular pressing (ECAP) [8,9,10] and related methods (twist channel angular pressing—TCAP [11], twist channel multi angular pressing—TCMAP [12], equal channel angular pressing-Conform—ECAP-Conform [13], equal channel angular pressing with partial back pressure—ECAP-PBP [14], etc.) can be considered to be very favourable. However, the majority of SPD methods are designed to process very small bulk samples. Laminates can also be produced by conventional forming methods (such as rolling, forging, drawing, and extrusion), but the amount of shear strain that can possibly be introduced by conventional methods is limited. Rotary swaging (RS) is a method of intensive plastic deformation, by which a high amount of shear strain can advantageously be imposed. Therefore, RS is a favourable method for the production of complex composites [15]. Among other advantages of RS is the dominating compressive stress state supporting metallic bonding of the individual layers [16], and its versatility (it can be used to process long axisymmetrical products—rods and wires [17]).

The applied processing conditions, such as the deformation ratio, stress state, and amount of imposed strain, affect the deformation behaviour of the laminate (formation of slip or kink bands, etc.), preferential deformation mechanisms (dislocation slip, dislocation climb, twinning, etc.), structure modifications (subgrains’ formation, texture orientations, grain size, etc.), and consequently also the mechanical, physical, electrical, and utility properties [18]. For example, inhomogeneous stress distribution, i.e., inhomogeneous distribution of residual stress, not only contributes to decreased quality or longevity of components’ bonding, but also deteriorates other properties, e.g., electric conductivity [19,20]. Nevertheless, the majority of the mentioned parameters can be optimized during processing.

Laminates typically consist of two (Al and Mg [21], Al and Ni [22], Al and Sn [23], Cu and Ni [24], Cu and Ti [25], etc.) or more (Cu and Al and Zn [26], Cu and Al and Mg [27], Cu and Al and steel [28], Al and Cu and Zn and Ni [29]) metallic materials (elements). Nevertheless, the most commonly researched and fabricated are materials consisting of combinations of Al and Cu. These two metals are popular components for laminated conductors, of which various designs and stacking sequences have been researched e.g., [3,15,18,21,25]. Laminates consisting of Al and Cu feature advantageous thermal and electric conductivity, favourable corrosion resistance, and lighter weight and lower cost when compared to (commercially) pure Cu [30]. Al/Cu composites are prone to form hard and brittle intermetallic phases at mutual interfaces. However, methods of plastic deformation performed at optimized processing conditions enable successful room temperature fabrication of Al/Cu laminates. Room temperature processing is also favourable from another viewpoint: During processing at elevated temperatures, both the Al and Cu are highly prone to dissolve oxygen. Dissolved oxygen significantly deteriorates the electric conductivity of Cu. Moreover, the presence of Cu_2_O precipitates in the structure substantially reduces plasticity and durability and can result in the formation of bubbles and subsequent cracking when in contact with hydrogen (i.e., water) [31].

Although works dealing with the fabrication of Al/Cu laminates via methods of plastic deformation have been published before, studies reporting the effects of processing procedures on interactions of (sub)structures and electric properties are scarce. Prospective applications of the presented laminated conductors are e.g., in the manufacture of rotors of asynchronous machines. The existing manufacturing technology of rotor windings involves (vacuum) die-casting, which introduces defects significantly deteriorating the overall quality of the windings (e.g., air bubbles, entrails, coagulants). For high-efficiency machines, the contemporary tendency is to shift from Al windings to Cu cages. However, the casting of Cu is a complex process performed at high temperatures, which can deteriorate its electrically insulating properties. Room-temperature fabrication of Al/Cu or Cu/Al composite conductors of the required final dimensions, which can be used directly for rotor bar windings of asynchronous machines, via swaging is thus very promising.

The presented work aimed to characterize the correlation between structure characteristics and electric behaviour for a uniquely designed Al/Cu laminated composite. The laminate was fabricated via room-temperature rotary swaging, and subsequently subjected to two different heat treatments. Before fabricating an electric conductor, it is favourable to determine the type of current that will be used during its testing and usage. In other words, will the conductor be used to transfer alternate or direct current (the transfer efficiency is affected by the occurrence of skin effect for alternate current)? The herein presented Al/Cu laminate is considered to be applicable for the transfer of both direct and alternate electric currents. However, the main focus of this study was to characterize the electric behaviour of the laminate during direct current transfer. The electric characteristics were measured on the experimentally swaged (and heat treated) laminated rods, and also numerically simulated. Structure analyses were performed via scanning and transmission electron microscopies. Microhardness measurements were performed to supplement the structure observations.

## 2. Materials and Methods

### 2.1. Experiment

**Figure 1:**
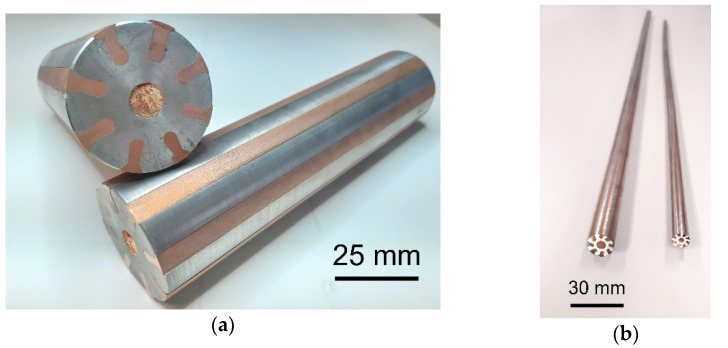
Al/Cu laminated workpiece (50 mm diameter) (**a**); swaged laminate (15 mm diameter) (**b**).

The materials used to produce the laminates were electro-conductive commercially pure (CP) Cu (Cu and 0.015% P, 0.002% Zn, 0.002% O), and electro-conductive CP Al (Al and 0.25% Fe, 0.21% Si, 0.04% Cu). The laminates were prepared by rotary swaging at room temperature (~23 °C). The unique stacking sequence of the Al/Cu laminate was designed based on our previous experience: The peripheral and axial regions of the laminate should consist of Cu, while the remaining volume of the laminate should consist of Al [32,33]. The Cu lamellas were located in a stellulate pattern within the Al matrix (the laminated workpiece is shown in the photo in Figure 1a). The diameter of the original composite workpiece, which was finally swaged down to the diameter of 15 mm, was 50 mm (the swaged laminated composite is shown in the photo in Figure 1b). The length of the original workpiece was 150 mm. The final reduction ratio for the laminate, calculated using Equation (1), was 2.4:

(1) $$\varphi =\ln\left(\frac{S_{0}}{S_{n}}\right)$$

where *S*_0_ and *S_n_* are laminate cross-section areas at the input and output from swaging dies, respectively.

The swaged products were further subjected to two annealing regimes to characterize the effects of post-process heat treatments on their structures and electric characteristics. The first regime was carried out at the temperature of 250 °C (regime *HT1*), and the second one was performed at the temperature of 350 °C (regime *HT2*). The post-process annealing treatments were selected considering the data acquired during our previous research—when subjected to post-process annealing, structures of the swaged laminates exhibited the tendency to recover. Nevertheless, annealing at temperatures higher than 350 °C imparted grain growth and the formation of intermetallics [34]. For each of the heat treatment regimes, the electric furnace was heated to the required temperature. Then, the laminate was inserted into the heated furnace for a dwell time of 15 min. After the time dwell, the laminate was taken out of the furnace and cooled freely on air.

Subsequently, the electric characteristics of all the swaged and heat-treated laminates were measured. Electric resistivity of the rods was detected experimentally using the highly accurate Four-Wire Resistance Measurements method. During the measurements by this method, two pairs of electrodes were used: Sense probes, which measured the voltage drop on the determined length of the laminate, and source probes, which supplied current to the measured laminate [35]. The use of two pairs of separate electrodes ensured that no supply current passed through the sensing electrodes, and that the sensing electrodes measured the voltage drop *V_1_* only. The source probes were connected in series with a calibrated shunt resistor to provide an accurate current measurement; the values of the current supplied by the used source were between 0 and 100 A. The voltage drop *V*_2_ monitored by the shunt resistor was directly related to the applied electric current via the *K* = 0.0004 Ω constant. Therefore, the current value could be characterized by the relation *I*_1_ = *V*_2_/*K*. The sense probes monitoring the voltage drop *V*_1_ during current flow were fixed on the measured laminate with the mutual distance *dL* = 500 mm. By using Ohm’s law, *Rs* (electric resistivity of the swaged laminate) on the measured length *L* (500 mm) was further computed by dividing the voltage drop *V*_1_ and electric current *I*_1_ as *Rs* = *V*_1_/*I*_1_.

Measurements of electric resistivity *Rs* of the swaged laminated rods should be carried out quickly since the conductors exhibit the tendency to heat by the effect of current flow. During the measurements, the current value increased rapidly from 0 A to 100 A, and then dropped again to 0 A. For such rapid changes, manual data recording was not possible. For this reason, both the voltage drops *V*_1_ and *V*_2_ were detected synchronously using a DAQ (Data AcQuisition) card (NI-9238 type) and stored in a data file. The DAQ card had a 24-bit resolution at the voltage range of ±500 mV. This enabled us to achieve the minimum measurable sensitivity *dU* = 60 nV. The stored data then provided the basis for the calculation of specific electric resistivity *ρ* via Equation (2),

(2) $$\rho =R\cdot \frac{S}{L}=\frac{V_{1}}{I_{1}}\cdot \frac{S}{L}=\frac{V_{1}}{I_{1}}\cdot C$$

where *R* is the resistivity [Ω], *S* is the cross-sectional area of swaged laminate [m^2^], *V*_1_ is the voltage drop [V], *I*_1_ is the DC current supply [A], *L* is the measured length between the voltage sensors of the swaged laminate [m], and *C* is the constant for the characteristic dimension [m].

**Figure 2:**
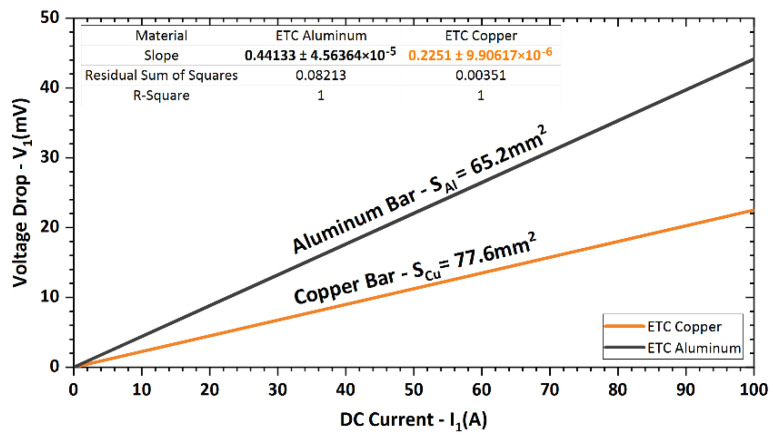
Measured voltage drop *V*_1_ in dependence on applied current *I*_1_ for original metals.

Before measuring the electric characteristics of the laminated composite rods, the data for the original Al and Cu materials were acquired. Figure 2 depicts the dependence of the voltage drop *V*_1_ on the electric current for the original Al and Cu laminate components. The measured values were smoothed by a linear fit for both metals. The slopes of the curves (*V*_1_/*I*_1_) determine the resistivity *R* of the measured metals per 1 m of length.

Structure analyses of the swaged (and heat treated) laminated rods were carried out by scanning and transmission electron microscopies (SEM and TEM). The samples for SEM electron backscatter diffraction (EBSD) observations, prepared from transversal cuts, were grinded manually, polished electrolytically, and observed using a Tescan Lyra 3 XMU FEG/SEMxFIB device equipped with a Symmetry EBSD detector (Tescan, Brno, Czech Republic). The analyses were carried out with the step of 0.5 µm on samples tilted by 70° and evaluated with the help of Aztec Crystal software (Oxford Instruments, Abingdon, UK). Detailed analyses of substructures of the Cu lamellas from the swaged and heat-treated laminates were performed using TEM (JEOL JEM-2100 device, JEOL, Tokio, Japan). The observations were performed at 200 kV on samples prepared using the focused ion beam (FIB) method assembled on the Tescan Lyra 3 XMU microscope. During FIB preparation, a lamella was milled with Ga ions in multiple steps to a final thickness of about 120 nm. The FIB lamella was taken from a transversal cut through a peripheral Cu lamella, and thus the TEM observations were carried out in a direction parallel to the swaging axis. The last experimental step involved Vickers microhardness measurements, which were performed using a Zwick/Roell testing device (Zwick Roell CZ s.r.o., Brno, Czech Republic). For each indent, the load was 200 gf, and the loading time was 10 s.

### 2.2. Numerical Analysis

To supplement the results of experimental investigations, numerical simulations of the current transfer were performed for the swaged laminated rods with diameters of 20 mm, 15 mm, and 10 mm. The geometries of the swaged rods models ensued from the experimentally observed ones. In other words, the locations and deformations of the individual composite components of the modelled rods were based on the locations and deformations of the Al/Cu laminate components observed experimentally via SEM.

**Figure 3:**
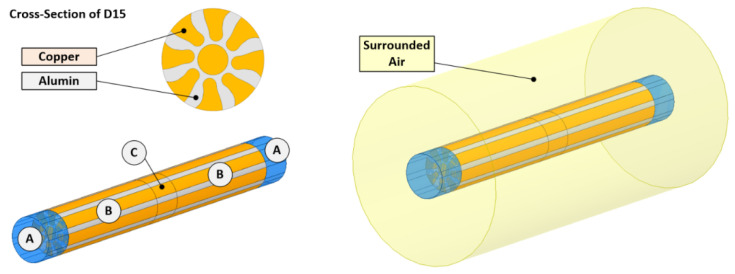
Model of laminated rod with individual components.

Figure 3 depicts a model of the laminated rod with individual modelling components (introduced for simulation purposes) depicted with the letters *A*, *B*, and *C*, and the cross-section of a laminated rod corresponding to the real laminate swaged to the diameter of 15 mm. The model of the laminated rod consisted of three individual modelling components:

- Components *A*—auxiliary components serving as the input and output of the electric current. The terminal cylindrical material volumes served to stabilize the current lines occurring by the effect of current transfer and represented the total current *I* = 100 A. In the numerical model, these terminal volumes were defined as conductors with negligible electric conductivity.
- Components *B*—represented the operational part of the laminated conductor. They enabled homogeneous transformation of the computational elements in the model and provided the smooth attachment of the central part of the laminate.
- Components *C*—Represented the significant (i.e., central) part of the laminate, on which evaluation of the output parameters was performed. The basic parameters were the distribution of the current density across the cross-section of the swaged laminate, and the power loss occurring due to the flowing current.

The electromagnetic model of the laminated conductor was used to perform a harmonic electromagnetic analysis using Ansys Electronic Desktop software. A harmonic electromagnetic simulation enabled us to determine the value of power loss dP (W) when the value of the current in the conductor was known. The value of the current used in the simulation, as well as during the experiments, was *I* = 100 A. After determining the values of transferring current (input parameter) and power loss (output parameter), the calculation of the total value of electric resistance of the laminated conductor was performed via Equations (3) and (4),

(3) $$dP=\frac{1}{\sigma }\overset{}{\underset{vol}{\int }}J^{2}dV$$

(4) $$R=\frac{dP}{I^{2}}$$

where *dP* is the current loss (W), *σ* is the electric conductivity of the material from which the conductor is fabricated (Sm^−1^), *J* is the current density (A/m^2^), *dV* is the element of the conductor volume (m^3^), *R* is the resistance of the conductor (Ω), and *I* is the value of the supply current.

**Figure 4:**
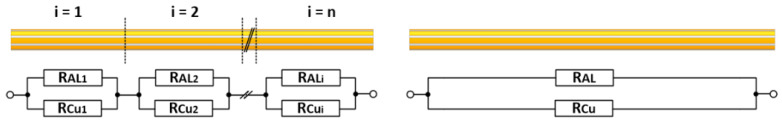
Schematic depiction of series and parallel circuits.

To determine the final value of electric resistance of the laminated rod, the basic equation for the calculation of the total resistance of net resistances in series and parallel circuits (see Figure 4) was used.

The parallel resistance parts, RAli||RCui, represented the length portion of the laminated conductor selected for the elements *i*. In the elements (nodes) *i* of the conductor, the ideal mutual connection between the laminate components was considered. The final resistance of the entire length of the conductor then consisted of the sum of resistances for the elements *i*, which represented the parallel combination of *n* resistances determined by the cross-section of the conductor, and specific electric resistivity of the relevant metallic component of the laminate. Element *i* of the laminate also represented 1/*n* portion of the total resistance of the total conductor length. For this reason, the resulting final resistance was equal to the parallel combination of resistances of the individual metallic components. The final relation applicable for the calculation of specific electric resistivity of the laminated conductor was then depicted via Equation (5),

(5) $$\frac{1}{\rho_{lam}}=\left(\frac{A_{Al}}{\rho_{Al}}+\frac{A_{Cu}}{\rho_{Cu}}\right)\cdot \frac{1}{A_{lam}}$$

where *ρ* is the specific electric resistivity (Ωm), *A* is the area (m^2^), *_Cu_* and *_Al_* are indices to characterize the individual component metals, and *_lam_* is the index to characterize the laminated rod.

## 3. Results

### 3.1. (Sub)structure Development

**Figure 5:**
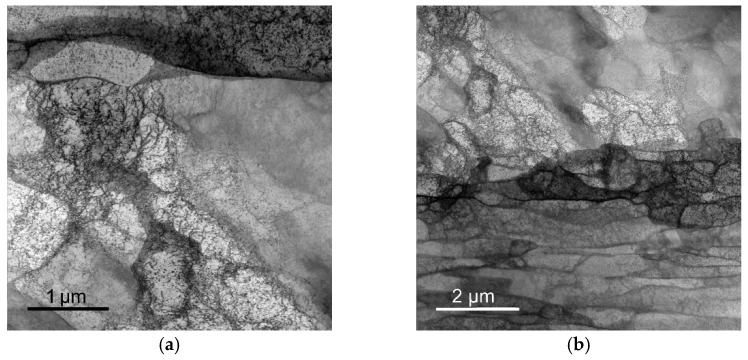

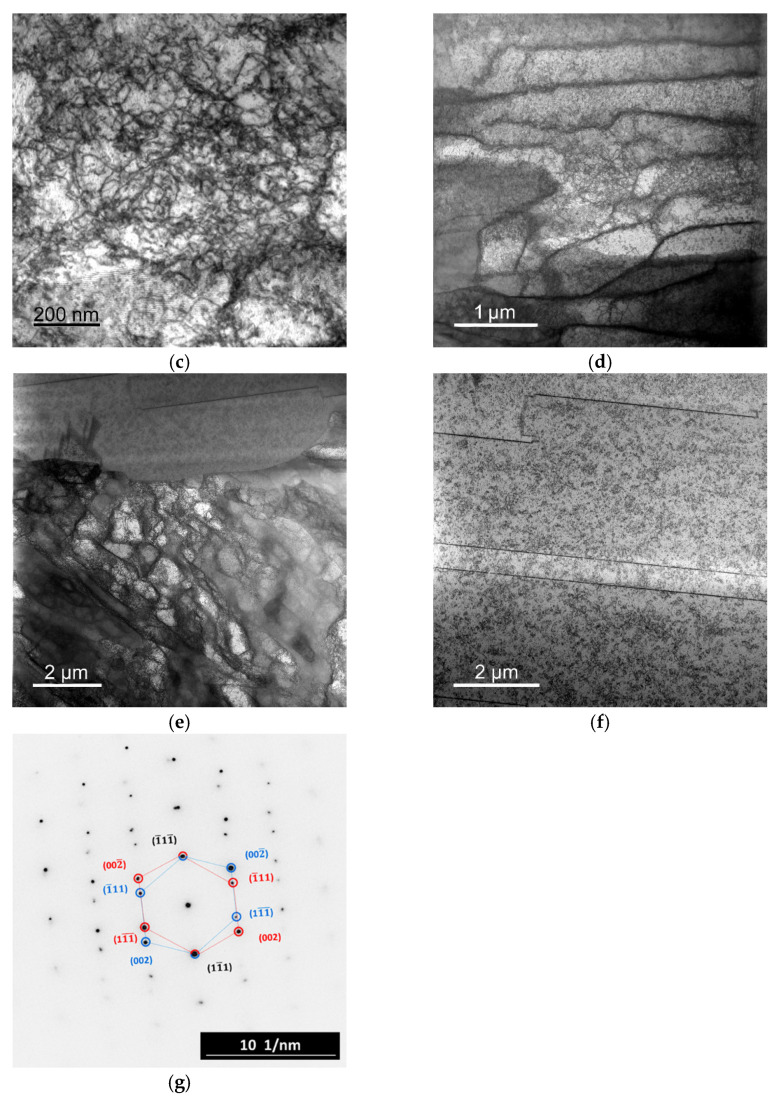
TEM images depicting characteristic structural features for Cu lamellas of swaged laminate (**a**,**b**); *HT1* laminate (**c**,**d**); *HT2* laminate (**e**). Twin within structure of *HT2* laminate (**f**) and its diffraction pattern (**g**).

Substructure developments within peripheries of the Cu lamellas of all the swaged (and heat treated) laminated rods were observed in detail via TEM. Swaging down to the diameter of 15 mm imparted the accumulation of structural defects and substructure formation. Accumulated dislocations forming dislocation cells within the swaged laminate are depicted in Figure 5a, while Figure 5b shows, in detail, evident deformations of grains (horizontal elongation, i.e., elongation in the axial direction of the laminate, imparted by the effect of axial component of the swaging force) and the formation of subgrains within. Heat treatment via regime *HT1* did not impart substantial restoration, as the structure still contained a high density of dislocations (Figure 5c depicts the interior of grain with accumulated dislocations). However, some grains already exhibited a decrease in dislocation density and the tendency to recover (Figure 5d). Finally, the *HT2* heat treatment imparted the structure recovery and annihilation of dislocations; the *HT2*-treated laminate still contained deformed grains, but also dislocation-free grains. Figure 5e depicts both the mentioned structural features and also documents the presence of the bimodal structure distribution within the *HT2*-treated rod. The *HT2* treatment also introduced the formation of twins. Figure 5f shows the detail of a restored grain featuring an annealing twin, of which the diffraction pattern is then shown in Figure 5g.

**Figure 6:**
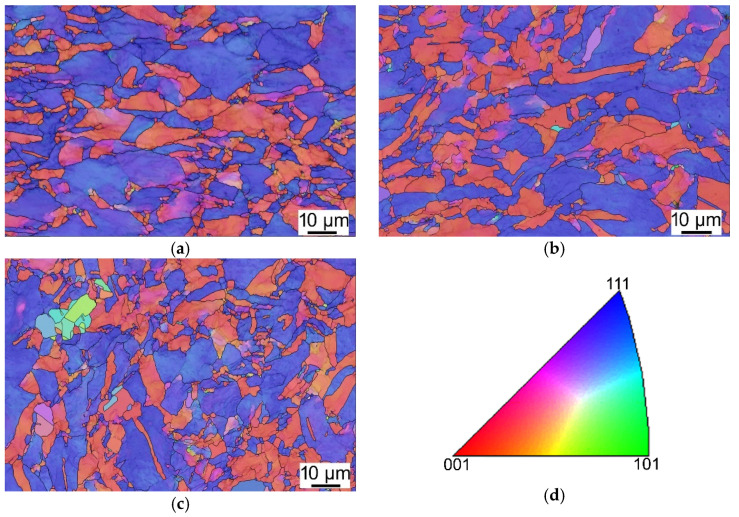
SEM OIM images for Cu lamellas of swaged laminate (**a**); *HT1* laminate (**b**); *HT2* laminate (**c**); OIM legend (**d**).

The results shown above of TEM observations supplemented the SEM analyses, which also showed heavily deformed grains within the Cu lamellas of the swaged laminate featuring the preferential orientations of the <001> and <111> || swaging direction (SD) (see Figure 6a depicting the orientation image map, OIM, for a Cu lamella of the swaged rod). Similar results of grains’ orientations were acquired for the *HT1* laminate (see the OIM in Figure 6b). However, this sample already exhibited a few relatively small, recrystallized grains with random preferential orientations (small, recrystallized grains diverging from the <001> and <111> || SD preferential orientations can be seen at the boundaries of larger original grains in Figure 6b). The Cu lamellas of the heat-treated laminate via regime *HT2* then exhibited an evident presence of recrystallized, randomly oriented grains at the boundaries of the original grains (see Figure 6c). The *HT2* sample also exhibited a bimodal grain size distribution and annealing twins (see Figure 6d depicting a larger area—compared to Figure 5f—featuring the bimodal grain size distribution and twins).

**Figure 7:**
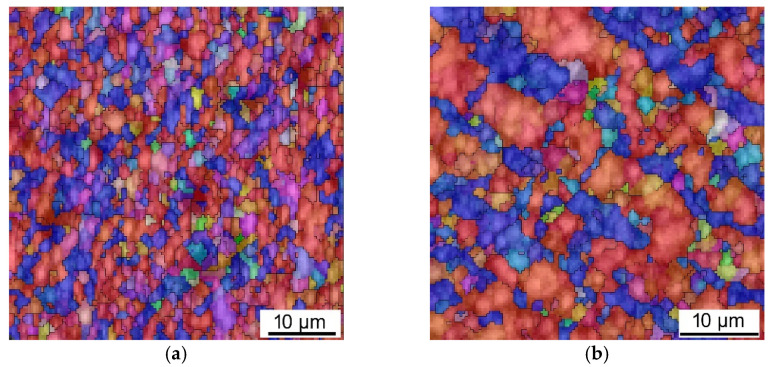

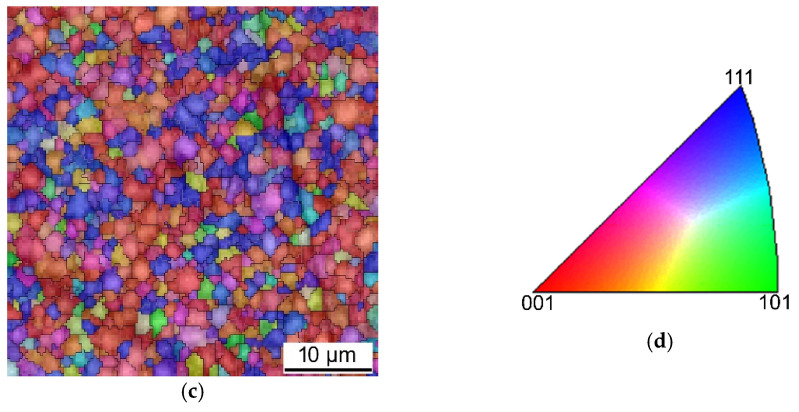
SEM OIM images for Al matrix of swaged laminate (**a**); *HT1* laminate (**b**); *HT2* laminate (**c**); OIM legend (**d**).

The SEM OIM images for the Al matrices of the swaged, *HT1-*, and *HT2*-laminated rods are depicted in Figure 7a–c, respectively. Similar to the further discussed grain size analyses (Section 3.2), the analyses of Al grains’ orientations did not reveal any significant differences between the individual material states. As the Al matrix most probably exhibited dynamic recrystallization during swaging to the final laminate diameter of 15 mm, all the Al components within the three examined laminates exhibited more or less equiaxed fine grains featuring comparable portions of the <001> || SD and <111> || SD preferential orientations. The portion of randomly oriented recrystallized grains within the Al components were generally higher than within the Cu components for all the investigated swaged (and heat treated) laminates.

### 3.2. Grain Size

The effects of the intensive plastic deformation on grain sizes of the components of the laminate were assessed, too. The grain sizes were evaluated using the maximum Feret diameter parameter, which is defined as the largest distance between two points defining an individual grain [36]. The average grain sizes for the components of the laminated rods were calculated from the overall numbers of grains present at the respective scanned regions. The graphical depictions of grain sizes were presented as the area-weighted fraction distributions, which depict sums of areas occupied by the grains with the respective diameter, i.e., total areas occupied by the grains at the scanned region, instead of the total number of grains with the respective size [37].

The average grain sizes (in µm) for the original Cu and Al were 36.9 µm and 64.0 µm, respectively. The average grain sizes within the swaged laminate were 3.1 µm for the Cu and 3.0 µm for the Al components. The *HT1* treatment imparted grain growth (especially for the Cu components), as the average grain sizes for the *HT1* laminate were 4.8 µm for Cu and 3.2 µm for Al components. Compared to the swaged state, the standard deviations from the average grain sizes also increased for both the metals, which confirmed the presence of a bimodal grain size distribution (as documented also in Section 3.1). Finally, the *HT2* treatment resulted in a slight decrease in the average grain sizes, as their values for the *HT2* laminate decreased to 4.2 µm for the Cu and 2.8 µm for the Al components. This fact could be attributed to the abovementioned increased fractions of small, recrystallized grains for this laminate; also, the standard deviations decreased again compared to the *HT1* laminate.

**Figure 8:**
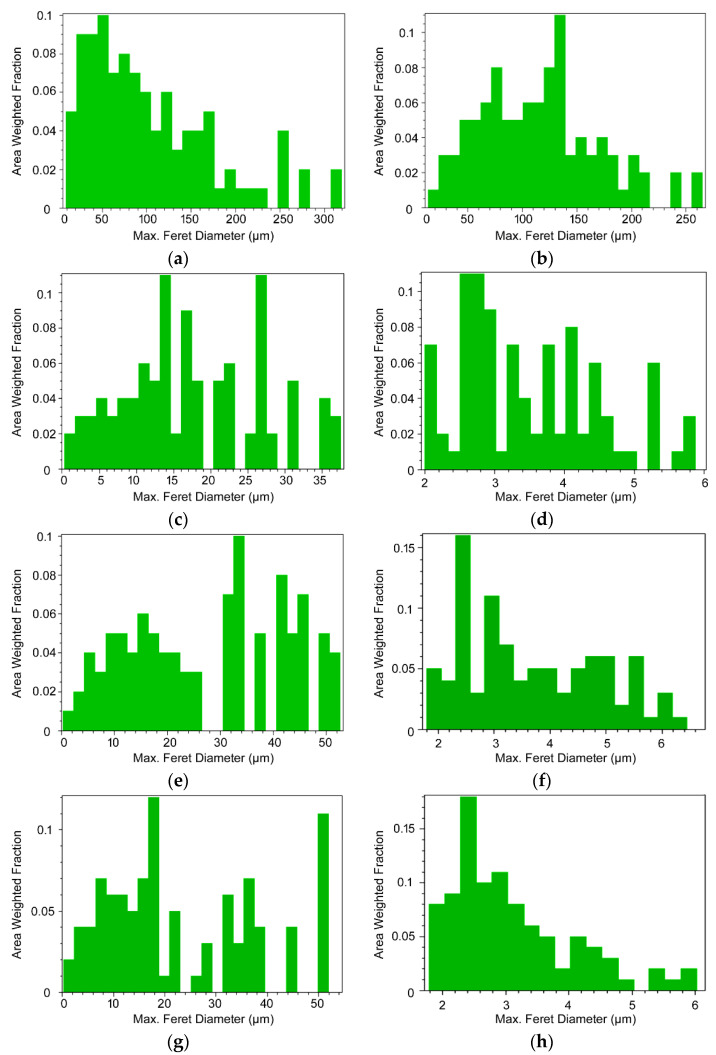
Grain size distributions for original metals: CP Cu (**a**), CP Al (**b**). Grain size distributions for swaged laminate: Cu lamella (**c**), Al matrix (**d**); *HT1* laminate: Cu lamella (**e**), Al matrix (**f**); *HT2* laminate: Cu lamella (**g**), Al matrix (**h**).

The grain size distributions for the original CP Cu and CP Al are depicted in Figure 8a,b, respectively. For the laminates, the analyses were performed at the peripheries of the swaged (and heat treated) rods, since the effect of the deformation processing was the highest in these regions [38]. Figure 8c,d depicts the area-weighted grain size distributions for the Cu lamella and Al matrix, respectively, of the swaged laminated rod. Figure 8e,f then shows the area-weighted grain size distributions for the Cu lamella and Al matrix, respectively, of the swaged laminated rod heat treated via regime *HT1*. Finally, Figure 8g,h depicts the area-weighted grain size distributions for the Cu lamella and Al matrix, respectively, of the swaged laminated rod heat treated via regime *HT2*.

### 3.3. Microhardness

**Figure 9:**
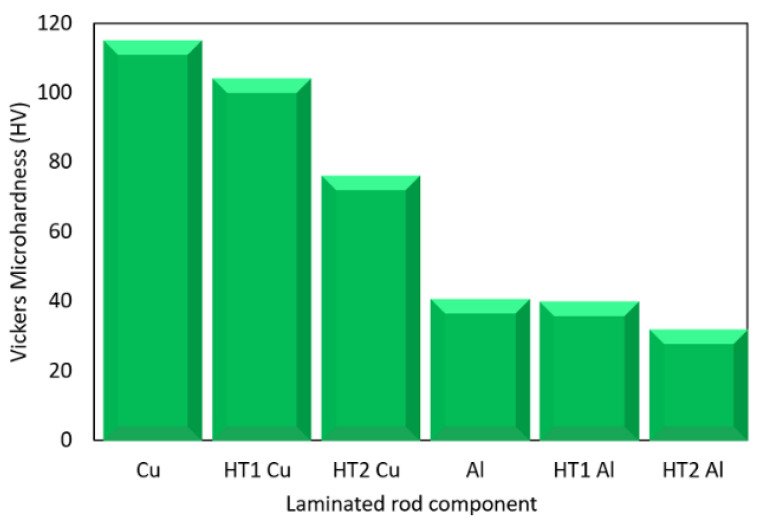
Measured Vickers microhardness values for laminates’ components.

As evident from Figure 9, the highest Vickers microhardness value of 115 HV was measured for the Cu lamellas of the swaged laminated rod. The microhardness of the Cu lamellas then decreased as a result of the effect of structure softening introduced by the post-process heat treatments; the lowest average microhardness value measured for Cu lamellas (76.5 HV) was detected for the lamellas of the HT2 rod. As regards the Al matrices, the differences between the microhardness values of the individual laminated rods were not as significant as for the Cu lamellas as the Al exhibited more significant dynamic recrystallization during swaging (documented in Section 3.1). The average HV values for the Al components of the swaged and HT1 rods were comparable (41 HV and 40.3 HV, respectively). The HV value then slightly decreased to 32.3 HV for the Al matrix of the HT2 rod.

### 3.4. Electric Properties

**Figure 10:**
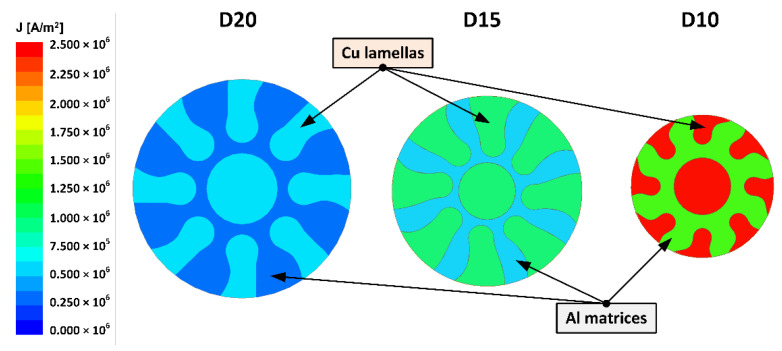
Numerically simulated distribution of current density across cross-sections of laminates swaged to 20 mm, 15 mm, and 10 mm (cross-sectional layouts of Al and Cu components based on geometries observed by SEM scanning of cross-sections of experimentally swaged laminates).

As regards the electric characteristics of the swaged (and heat treated) laminates, the results of the numerical simulations were evaluated at first. The basic output of the harmonic analysis was the distribution of current density across the cross-sections of the swaged laminates, which is depicted in Figure 10 for the laminates swaged to the diameters of 20 mm, 15 mm, and 10 mm (the geometrical layout of the individual metallic components across the laminates’ cross-sections corresponded to the real geometries of the laminates observed by SEM). As evident from the figure, the current density was distributed homogeneously across the cross-sectional areas of all the examined laminates. Higher current density values were observed in the axial Cu cores, as well as in the peripheral Cu lamellas. In other words, generally lower current density values were observed for the Al matrices, whereas higher values were observed for the Cu lamellas.

The methods by which the electric characteristics of the laminates were further experimentally measured and calculated were described in Section 2, in which the results of measurements of electric resistivity for the CP metals were also depicted. Expressed in numbers, the specific resistivity and resistance, respectively, were 17.468 Ωm × 10^−9^ and 225.1 Ω × 10^−6^ for Cu, and 28.772 Ωm × 10^−9^ and 441.3 Ω × 10^−6^ for Al. During evaluations of the electric properties of the laminates, these values of the original metals were considered.

**Figure 11:**
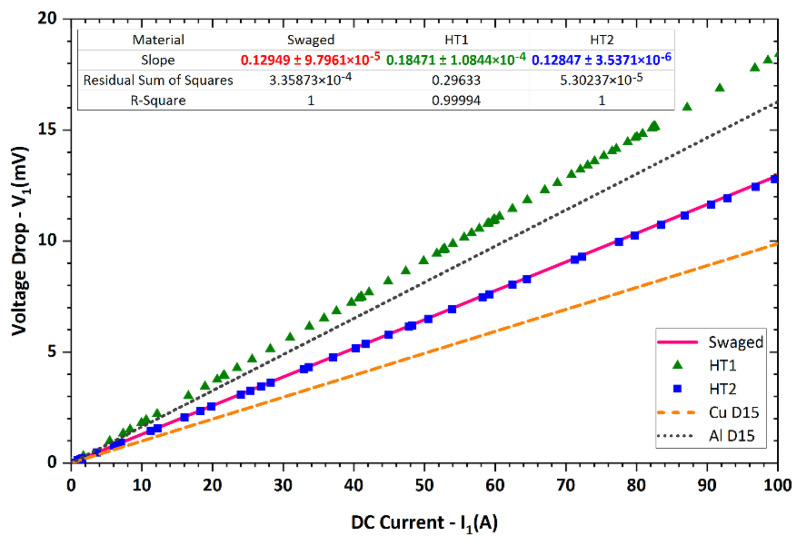
Experimentally acquired dependences of voltage drop on applied direct current for swaged and heat-treated laminates and for swaged original metals.

Figure 11 depicts the dependence of the voltage drop *V*_1_ on the applied electric current for the swaged (and heat treated) laminated rods. Similar to the original CP metals, the data for the laminates were smoothed by linear fits. The figure thus depicts that the values of the voltage drop exhibited a linear increase with increasing electric current applied for all the examined material states. The slopes of the curves again characterized the resistances *R* of the laminated rods. As can be seen from the figure, the resistances of the swaged laminate and laminate heat treated via regime *HT2* were comparable. However, the laminate heat treated via regime *HT1* exhibited significantly increased resistance. To enable a comparison of resistances of the produced laminates, Figure 11 also includes the curves acquired experimentally for CP Al and CP Cu rods, i.e., rods of the original metallic components swaged to a diameter of 15 mm. Evidently, the electric conductivity of the swaged laminate was superior to that of CP Al but deteriorated when compared to CP Cu. Similar results were also acquired for the *HT2* laminate. The *HT1* laminate exhibited deteriorated electric conductivity when compared to both the original metals in swaged states.

## 4. Discussion

The assembled laminated semi-products with an original diameter of 50 mm were gradually swaged to laminated rods with a diameter of 15 mm, which not only affected the structures of the laminates, but also their electric properties. Swaging with a total reduction ratio of 2.4 resulted in deformation strengthening and significant grain refinement, pointing to the occurrence of dynamic recrystallization during processing.

Generally, the grains within the Cu lamellas were larger than the grains within the Al matrices for all the swaged and heat-treated laminated rods. The primary reason for this phenomenon was that deformation strengthening was processed differently within the metallic components, which was given by the differences in their intrinsic properties and lattice parameters (the Al matrix tended to consume the imposed shear strain more easily than Cu) [31]. The Al matrices exhibited higher portions of recrystallized grains featuring more randomized orientations than the Cu lamellas. This fact, together with the abovementioned grain size results, points to the significant occurrence of dynamic recrystallization within the Al matrix during swaging. The energy imparted by the *HT1* treatment performed at 250 °C introduced the slight growth of the grains, rather than further recrystallization. This fact was also confirmed by the grain size measurements, as the average grain sizes of both the Al and Cu components increased slightly after the *HT1* treatment (compared to the swaged laminate). Nevertheless, increasing the annealing temperature to 350 °C imparted sufficient energy for the grains to exhibit the annihilation of dislocations (confirmed by TEM) and partial recrystallization, which was proven not only by the grain size analyses, but also by the presence of fine grains featuring randomized orientations (i.e., deviating from the <001> || SD and <111> || SD preferential orientations) within the Al matrix and Cu lamellas of the *HT2*-laminated rod.

The observed grain size changes were non-negligibly connected to the substructure development and changes in microhardness of the metallic components, as these phenomena were introduced by the imposed shear strain, i.e., deformation strengthening. Compared to the swaged state, microhardness decreased for the *HT2* laminate, especially due to the observed decrease in dislocation density, which resulted in structure softening. The structural phenomena occurring also introduced changes in the geometrical distribution of the metallic components across the cross-section of the laminated rod. As documented by the results of numerical simulations, the current density, as well as the consequent electric characteristics of the laminate, were not only affected by the area fractions of the metallic components across the cross-section of the laminate, but also by the electric conductivities of both the metallic components. The material featuring a lower electric resistivity, i.e., higher conductivity, had a higher tendency to transfer the electric current despite the fact that its cross-sectional area fraction across the transversal cut through the laminate was lower. The higher current density of the respective metallic component caused the electric current to flow primarily through that part of the laminated conductor featuring a lower value of specific electric resistivity. Moreover, from the viewpoint of the transfer of electric current through the laminated conductor, it was not important whether the lengths of the individual material components were mutually insulated, or closely connected, or whether they exhibited certain transitional resistance.

Figure 11 characterizing the experimentally observed electric behaviours of the laminated rods showed that all the swaged and heat-treated laminates exhibited deteriorated electric conductivity when compared to a swaged Cu rod. This fact was related to the above-discussed structural phenomena, i.e., to deformation strengthening of both the metallic components ensuing from grain size decrease, substructure development, and increase in dislocation density introduced by the intensive plastic deformation [39]. Interestingly, the values of voltage drop *V*_1_ measured for the commercially available electro-conductive CP Cu and CP Al components were different than those calculated for swaged CP Cu and CP Al rods (linearly increasing trend was considered). In other words, the *V*_1_ value at the maximum current of 100 A was *V*_1_ *=* 44 mV for Al and *V*_1_ *=* 22 mV for Cu (see Figure 2). Nevertheless, for rods swaged to the diameter of 15 mm, the theoretically calculated *V*_1_ values are *V*_1_ = 26.5 mV for CP Al and *V*_1_ = 14.6 mV for CP Cu. However, the real values measured for the 15 mm rods swaged within this study were *V*_1_ = 16 mV for CP Al and *V*_1_ = 10 mV for CP Cu (see Figure 11). This fact confirmed that swaging imparted significant positive changes in the microstructures of both the metallic components (compared to original annealed CP states). The imposed strain imparting the substructure development thus favourably affected electric conductivity, i.e., resulted in an electric resistivity decrease. Generally, the presence of annealing twins, which is typically significant in rolled/drawn and annealed Cu pieces, contributes to an electric resistivity increase [39]. This was most likely the primary reason for the higher resistivity of the original annealed CP Cu (compared to the swaged CP Cu rod). On the other hand, the larger grain size, which is typically observed within annealed non-deformed metals, contributes to higher electric conductivity given by the relatively low volume of grain boundaries, which act as barriers during the electric current transfer (similar to other structural features, which can possibly be present in deformed structures, such as dislocation cells and subgrains).

Partial structure restoration, i.e., the presence of small, recrystallized grains with randomized preferential orientations within the structure (primarily the Al matrix), imparted an increase in the electric resistivity. In other words, partial reorientations of subgrains, i.e., grains defined by low-angle grain boundaries newly developing within the original grains [40], introduced by the heat treatment at the lower temperature (*HT1*) resulted in increased electric resistivity compared to the swaged material state. On the contrary, heat treatment at the higher temperature (*HT2*) imparted the annihilation of dislocations, the development of twins, and a relatively large fraction of recrystallized grains featuring randomized orientations. These phenomena again resulted in decreased electric resistivity—its value for the *HT2* laminate was comparable to that of the swaged laminate. In summary, structural phenomena, such as deformation twins, can decrease electric conductivity (particularly for pure metals). However, their presence in deformed laminates is not as significant from the viewpoint of increasing electric resistivity. On the other hand, heterogeneity in the orientations of (sub)grains can be a much more substantial factor influencing the electric conductivity of the laminate.

## 5. Conclusions

This paper aimed to characterize the electric properties and structural features of Al/Cu-laminated rods, which were successfully prepared via combinations of room-temperature rotary swaging and post-process heat treatments. The assembled composites 50 mm in diameter were progressively swaged down to laminated rods with a diameter of 15 mm, and subsequently subjected to two heat treatment regimes (250 °C = *HT1,* and 350 °C = *HT2*). The main acquired results were the following:

- Swaging to the diameter of 15 mm imparted favourable electric characteristics and an increase in microhardness up to 115 HV for the Cu components of the laminate.
- *HT1* material state—the occurrence of relatively small, restored grains with randomized preferential orientations was the main reason for the observed deterioration of electric conductivity (specific electric resistivity of approx. 34 × 10^−9^ Ωm compared to approximately 23.5 × 10^−9^ Ωm for the swaged state).
- *HT2* material state—the electric conductivity improved to a level comparable with the swaged state (approximately 22.7 × 10^−9^ Ωm); in addition, the higher annealing temperature caused bimodal grain size distribution and the development of annealing twins.
- A favourable effect of rotary swaging on the electric conductivity was also observed for the original Al and Cu (theoretically calculated voltage drop values were higher than experimentally measured for both)—this phenomenon was related to substructure development (substantial grain refinement and high dislocation density) imparted by swaging.

The presented results proved that the Al/Cu laminate of the proposed stacking sequence is suitable for the production of conductors for direct current transfer. Although there are differences between the characteristics of alternate and direct currents (primarily the occurrence of the skin effect during alternate current transfer), the Al/Cu laminate is supposed to also be suitable for alternate current transfer; the detailed investigation of the behaviour of the Al/Cu laminate during alternate current transfer is the primary focus of our ongoing research.

## Data Availability

The original data supporting the research are not publicly available but the data that are not confidential are available on request from the corresponding author.
